# Provider and community stakeholder perspectives of expanding Medicaid coverage of abortion in Illinois

**DOI:** 10.1186/s12913-022-07761-5

**Published:** 2022-03-28

**Authors:** Carmela Zuniga, Aalap Bommaraju, Lee Hasselbacher, Debra Stulberg, Terri-Ann Thompson

**Affiliations:** 1 Ibis Reproductive Health, 2067 Massachusetts Ave, Suite 320, Cambridge, MA USA; 2 Department of Sociology, University of Cincinnati, Crosley Tower, 301 Clifton Court, Cincinnati, OH 45221-0378 USA; 3 Center for Interdisciplinary Inquiry and Innovation in Sexual and Reproductive Health (Ci3), University of Chicago, 6030 South Ellis Avenue, Chicago, IL 60637 USA; 4 Department of Family Medicine, University of Chicago, 5841 South Maryland Avenue, MC7110, Room M156, Chicago, IL 60637 USA

**Keywords:** Abortion, Medicaid, Illinois, Insurance, Hyde amendment, Abortion access

## Abstract

**Background:**

Many people seeking abortion encounter financial difficulties that delay or prevent them from accessing care. Although some patients qualify for Medicaid (a public program that can help cover health care costs), laws in some states restrict the use of Medicaid for abortion care. In 2017, Illinois passed House Bill 40 (HB-40), which allowed patients with Medicaid to receive coverage for their abortion. This study aimed to understand how HB-40 affected abortion affordability from the perspectives of individuals that work directly or indirectly with abortion patients or facilities providing abortion care.

**Methods:**

We conducted interviews with clinicians and administrators from facilities that provided abortion services; staff from organizations that provided resources to abortion providers or patients; and individuals at organizations involved in the passage and/or implementation of HB-40. Interviews were audio-recorded and transcribed. We created codes based on the interview guides, coded each transcript using the web application Dedoose, and summarized findings by code.

**Results:**

Interviews were conducted with 38 participants. Participants reflected that HB-40 seemed to remove a significant financial barrier for Medicaid recipients and improve the experience for patients seeking abortion care. Participants also described how the law led to a shift in resource allocation, allowing financial support to be directed towards uninsured patients. Some participants thought HB-40 might contribute to a reduction in abortion stigma. Despite the perceived positive impacts of the law, participants noted a lack of public knowledge about HB-40, as well as confusing or cumbersome insurance-related processes, could diminish the law’s impact. Participants also highlighted persisting barriers to abortion utilization for minors, recent and undocumented immigrants, and people residing in rural areas, even after the passage of HB-40.

**Conclusions:**

HB-40 was perceived to improve the affordability of abortion. However, participants identified additional obstacles to abortion care in Illinois that weakened the impact of HB-40 for patients and required further action, Findings suggest that policymakers must also consider how insurance coverage can be disrupted by other legal barriers for historically excluded populations and ensure clear information on Medicaid enrollment and abortion coverage is widely disseminated.

**Supplementary Information:**

The online version contains supplementary material available at 10.1186/s12913-022-07761-5.

## Introduction

In the United States, approximately 75% of abortion patients earn low-incomes and the majority (65%) live below the federal poverty level [1]. For these abortion patients, insurance coverage for this essential reproductive service makes all the difference. One study found that the cost of an abortion is a catastrophic health expenditure (constituting 40% or more of a household’s monthly income after basic needs have been met) for households earning their state’s median income or less, suggesting that some households considered middle-income would struggle to afford an abortion if paid out-of-pocket [2]. Without insurance coverage, many people seeking abortion will find the average $465 out-of-pocket cost of a first trimester abortion to be a significant or insurmountable barrier [3, 4]. Moreover, the time spent gathering enough money for a first trimester abortion may push some pregnant people into needing a more expensive second trimester abortion, and the average out-of-pocket cost of $1000 for that procedure may prevent some from obtaining abortion care at all [3, 4].

Although low-income earners may qualify for Medicaid—a joint federal and state government program that assists low-income earners with health care costs—many cannot use this public insurance plan to cover the cost of their abortion due to federal and state policies. At the federal level, the Hyde Amendment prevents federal funds from covering abortion costs unless the pregnancy resulted from rape or incest, or endangers the life of the pregnant person. Although states can use their own funds to cover the cost of abortion for Medicaid recipients beyond those three reasons, only 16 do so as of May 2021 [5]. With the passage of House Bill 40 (HB-40) in 2017, Illinois became one of these 16 states to cover abortions using state Medicaid funds. HB-40 has the potential to impact the 1.5 million adults in Illinois enrolled in Medicaid [6]. HB-40 could also impact people eligible for Medicaid; roughly a third of an estimated 231,000 uninsured women of reproductive age were eligible for coverage through Medicaid or the Children’s Health Insurance Program (CHIP) based on their income in 2017 [7].

While research on affordability—one domain of access [8]—within contexts where Medicaid coverage is restricted is robust, research on affordability in contexts where Medicaid has been expanded is limited. One way to understand affordability is to learn from stakeholders with direct experience of supporting people seeking abortion. The implementation of HB-40 presents an opportunity to generate much-needed evidence on how increased affordability relates to abortion utilization and the complexities of abortion access.

In this study we aimed to understand how HB-40 affected the affordability of abortion care, thereby impacting utilization, from the perspectives of abortion providers and abortion access supporters that work directly or indirectly with abortion patients or facilities providing abortion care. We call this latter group non-providers. Through our analysis we highlight persistent gaps preventing individuals earning low incomes from utilizing Medicaid for abortion care and factors that may mediate the relationship between affordability and abortion utilization.

## Methods

Between March 2019 and March 2020, we invited 53 individuals to talk about their perspectives and experiences related to HB-40. We used a phenomenological study design and purposive sampling to collect perceptions of HB-40’s implementation from a wide array of stakeholder standpoints. These included clinicians and administrators in abortion facilities (providers), as well as staff in organizations that support abortion patients (non-providers). All potential research subjects were involved in HB-40’s passage and/or implementation. Providers were recruited from a sample representing all known clinics providing abortion during the first year that HB-40 took effect (January 1, 2018 - December 31, 2018), *n* = 25. Non-providers were recruited from a sample of organizations providing support to abortion patients and/or involved in HB-40’s passage and/or implementation, *n* = 18. We used personal and professional contacts, as well as snowball sampling to contact participants in each organization, inviting as many individuals as possible from the two groups. Most participants were known professionally to at least one of the authors. We recruited participants until data saturation (defined as the point at which no new themes emerged) [9] was reached.

Data was collected by the first and second authors, researchers at a non-profit and academic institution; who use she and they pronouns (respectively) and hold a masters and doctoral candidacy (respectively). Between April 2019 and April 2020, the first and second authors (both with 5 years of qualitative research experience) conducted in-person or phone interviews with 23 providers. A detailed description about the types of clinics we recruited can be found in a separate study [10]. Between October 2019 and April 2020, the second author conducted in-person or phone interviews with 15 non-providers from organizations that provided abortion-related resources or were involved in HB-40’s passage and/or implementation. The 38 interviewed participants represent 15 abortion clinics and 14 organizations that provided support to abortion patients and/or were involved in HB-40’s passage and/or implementation. Participants were informed about the study aims and the interviewer’s affiliation during recruitment, through the consenting process, and again during the interview. Interviews lasted less than 100 min, were completed only once, and did not include parties outside of the researcher and participants. Interviewers took notes during the interviews. Participants were not asked to review their transcripts or interview notes, nor provide feedback on the findings.

Although this paper focuses on interviewees’ perspectives of HB-40’s impact on patients, participants also answered questions about their perspectives on the consequences of the law for providers, as well as the law’s impact on local and national politics related to abortion access. The interview guides were developed by the research team (all authors listed) and was based on abortion affordability literature with special attention paid to coverage and operational processes [11, 12]. Questions related to the legislative process were drawn from the sociological literature on the role of advocacy organizations in U.S. political processes [13]. The interview guide for providers working at abortion facilities included three subject areas: insurance coverage (e.g. billing, reimbursement, and operational processes), public funding for abortion, and experience with HB-40’s passage (e.g. changes in population and services since the policy change and perspectives on the impact of Medicaid coverage for abortion). While the interview guide for non-providers covered: general knowledge and impressions of HB-40, experience with HB-40’s passage (e.g. changes in population and services since the policy change and perspectives on the impact of Medicaid coverage for abortion), and their impressions of other insurance changes since HB-40. While the interview guides were not tested prior to implementation, several of the questions were effectively used in previous studies on Medicaid coverage of abortion and abortion legislative processes. The guides are included as an Additional File.

Interviews were digitally recorded and a third-party transcription company transcribed all audio files verbatim. We used the Framework Method [14] to thematically analyze the transcripts and a combined (deductive and inductive qualitative) approach was used for thematic analysis. An initial list of thematic codes was developed based on the semi-structured interview guides and existing literature on abortion and abortion access in the United States. During coding, inductive codes were assigned to data that described a new theme. We did not use hierarchical coding to analyze the data, thus no coding tree was produced.

Two codebooks (one based on each guide) with definitions for each code was created. All transcripts, codes, and code definitions were uploaded to the web application Dedoose. We first analyzed transcripts from interviews with providers from abortion facilities. The first, second, and third authors began analysis by independently coding one transcript and met afterwards to compare coding methodology to ensure codes were applied consistently, and to refine or add codes to the codebook. The authors then independently coded a second transcript with the revised codebook and met again to compare coding strategies. As there were no major discrepancies between each author’s coding strategies, the first and third author then split the remaining transcripts to code. As new themes emerged from a transcript, the authors met to discuss the addition or revision of a code and applied these changes to transcripts that had previously been coded. The two authors split the organized data between themselves to analyze and summarize findings by code. The first author, second author, and a research assistant followed this same process to code and analyze the non-providers transcripts.

To protect the confidentiality of interviewees, we attribute each quote in this paper to the participant using a two-letter pseudonym that bears no relationship to their real name, and by a short description of the role their organizations played in relation to abortion care in Illinois (abortion provision, resource provision, or policy involvement). Participants received a $60 gift card for their participation in the study.

## Results

Of the 53 individuals invited to participate in the study, 38 completed interviews, 6 declined to participate, and 9 did not respond to the invitation. Of the 38 participants interviewed, 36 provided their age, which ranged from 22 to 71 years; the median age was 40.5 years. Table 1 summarizes other participant characteristics.

**Table 1:** Participant characteristics

	N	% Total
**Race/Ethnicity**		
Asian	5	13%
Black	4	11%
White	28	74%
Latin	1	3%
**Gender**		
Cis-Gender Woman	35	92%
Cis-Gender Man	3	8%
**Sexuality**		
Declined	3	8%
Heterosexual	27	71%
Lesbian, Gay, Bisexual, or Queer	8	21%
**Residential location**		
Chicago Area	25	66%
Declined	3	8%
Downstate Illinois	5	13%
Outside of Illinois	5	13%
Participants’ organizational affiliation (categorized by organization’s role related to abortion)*		
Provides abortion services	24	63%
Provides resources to providers or patients	5	13%
Involved in HB-40’s passage or implementation	9	24%

^a^Specific organizations may be represented more than once since some interviewees worked at the same organization

We grouped findings under three broad themes: perceived mechanisms connecting increased affordability to abortion utilization, perceptions of persistent barriers to abortion utilization, and factors that could diminish the impact of HB-40 on the affordability of abortion care. Table 2 presents illustrative quotes by themes.

**Table 2:** Illustrative quotes by themes

Themes	Illustrative quotes
Positive impacts of HB-40 on abortion access	
*Reduced financial stress*	"I can say that a patient that–and before the passage of the bill, would’ve had to find multiple resources, if any, to come up with some portion of the fee. And then, they would’ve had to kind of work with other funding agencies to see if they qualify, based upon the funding agencies requirements or qualifications, to see if they can get any financial assistance. And if they made it through all those hoops, they would get the services. But if not, they would not be able to seek abortion care."(SH, abortion provision)
	“The vast majority of people are just incredibly grateful that they […] are able to access [abortion] services without a significant amount of difficulty in terms of the cost.” (YV, abortion provision)
	"No out-of-pocket, no having to coordinate care with anything. You just have to have your active Medicaid, and the patients can just have their abortion paid for by their Medicaid, which is the right way to do it. But previously they could get some funding, but they generally – unless their situation was very extreme – had to come up with some on their own. And they had to figure it out, usually in advance, and do a little extra legwork, et cetera, and now it’s better" (ED, abortion provision)
	“It's more like an indirect benefit because […] we'll be able to give a fund to somebody who's undocumented if they didn't have to also give that fund to somebody who could use Medicaid […] so I think there's […] now more resources for people who have less access.” (LV, policy involvement)
	R: So we started definitely funding a lot more out-of-state folks. Yeah. But they had always been calling. And also, with the increased barriers in surrounding states – I don’t want to leave that out. You know Indiana is a mess, so the surrounding states have really – they’re stricter so – I: Right. So it wasn’t like there were more calls, it was just like you could – R: Yeah, I want to say it was like – yeah, because you couldn’t come and use your Medicaid here. You couldn’t use your form of insurance, your Medicaid insurance in Illinois, so they weren’t coming because of HB 40, it’s just it changed the dynamics in how we funded (NH, resource provision) R: So we started definitely funding a lot more out-of-state folks. Yeah. But they had always been calling. And also, with the increased barriers in surrounding states – I don’t want to leave that out. You know Indiana is a mess, so the surrounding states have really – they’re stricter so – I: Right. So it wasn’t like there were more calls, it was just like you could – R: Yeah, I want to say it was like – yeah, because you couldn’t come and use your Medicaid here. You couldn’t use your form of insurance, your Medicaid insurance in Illinois, so they weren’t coming because of HB 40, it’s just it changed the dynamics in how we funded (NH, resource provision) R: So we started definitely funding a lot more out-of-state folks. Yeah. But they had always been calling. And also, with the increased barriers in surrounding states – I don’t want to leave that out. You know Indiana is a mess, so the surrounding states have really – they’re stricter so – I: Right. So it wasn’t like there were more calls, it was just like you could – R: Yeah, I want to say it was like – yeah, because you couldn’t come and use your Medicaid here. You couldn’t use your form of insurance, your Medicaid insurance in Illinois, so they weren’t coming because of HB 40, it’s just it changed the dynamics in how we funded (NH, resource provision) R: So we started definitely funding a lot more out-of-state folks. Yeah. But they had always been calling. And also, with the increased barriers in surrounding states – I don’t want to leave that out. You know Indiana is a mess, so the surrounding states have really – they’re stricter so – I: Right. So it wasn’t like there were more calls, it was just like you could – R: Yeah, I want to say it was like – yeah, because you couldn’t come and use your Medicaid here. You couldn’t use your form of insurance, your Medicaid insurance in Illinois, so they weren’t coming because of HB 40, it’s just it changed the dynamics in how we funded (NH, resource provision)
	“In the beginning for the first three or four months women would show up because they’d already been scheduled before HB-40 and so they would have their money ready to pay. And we got to tell them that we didn’t need that money. That it was now gonna be covered by their insurance. And so we definitely had people crying and saying how happy they were. And how now they could pay their rent or buy food for their kids.” (OZ, abortion provision)
	“I think the perception, say, from the public – the public’s perception and from lots of people is that everything’s just great. And from the patients’ perspective, as well, which that is great that patients feel like it’s fixed and they can come get their abortion and they don’t have to worry about taking money away from the other necessities of life.” (YV, abortion provision)
	TF: Yeah. And people are shocked when they call, email, come in. They’re like, wait, how much do I have to pay? I’m like, no, nothing. They’re like, wait a minute, how much? MD: Yeah. It’s like, no, no, no, your copay is zero. Wait, what? We’re – TF: They’re literally sitting and like – [...] TF: Oh, my god, it’s how it should be. MD: I mean, people cry about that. I mean, they get very – TF: Oh, my – they’re like, thank you, so much. (MD and TF, abortion provision) TF: Yeah. And people are shocked when they call, email, come in. They’re like, wait, how much do I have to pay? I’m like, no, nothing. They’re like, wait a minute, how much? MD: Yeah. It’s like, no, no, no, your copay is zero. Wait, what? We’re – TF: They’re literally sitting and like – [...] TF: Oh, my god, it’s how it should be. MD: I mean, people cry about that. I mean, they get very – TF: Oh, my – they’re like, thank you, so much. (MD and TF, abortion provision) TF: Yeah. And people are shocked when they call, email, come in. They’re like, wait, how much do I have to pay? I’m like, no, nothing. They’re like, wait a minute, how much? MD: Yeah. It’s like, no, no, no, your copay is zero. Wait, what? We’re – TF: They’re literally sitting and like – [...] TF: Oh, my god, it’s how it should be. MD: I mean, people cry about that. I mean, they get very – TF: Oh, my – they’re like, thank you, so much. (MD and TF, abortion provision) TF: Yeah. And people are shocked when they call, email, come in. They’re like, wait, how much do I have to pay? I’m like, no, nothing. They’re like, wait a minute, how much? MD: Yeah. It’s like, no, no, no, your copay is zero. Wait, what? We’re – TF: They’re literally sitting and like – [...] TF: Oh, my god, it’s how it should be. MD: I mean, people cry about that. I mean, they get very – TF: Oh, my – they’re like, thank you, so much. (MD and TF, abortion provision)
	I think it's great that Medicaid is treating abortion like other services that are part of the spectrum of reproductive health care [...] Knowing that pregnancies that are unplanned can be very harmful to a woman's course of life in what she wants to be doing. And particularly, for low-income women, that can be something that could be financially devastating. And especially if they're not ready to have a child or have another child or for whatever reason." (FK, abortion provision)
	" I was happy, because it will help a lot of people. [...] It would make it less stressful on the patient, less stressful on the physician, and less stressful on the facility because we know we can provide good care for them. They’re getting what they need, so they’re gonna leave happy." (KK, abortion provision)
*Patient-driven care (choice of abortion method)*	I'm sure over time you'll see people who are getting abortions earlier, they just don't have to delay it as long in order to get them." (HW, policy involvement)
	But we are definitely seeing patients – we are seeing a huge increase in the medical abortions. I think part of that is because we’re seeing patients at much earlier gestational ages. And I don’t have the exact numbers on the gestational ages, but I could get you numbers on the percent changes in medical versus surgical abortions. But we’re seeing patients earlier. [...] “I think it’s because of Medicaid. They’re not waiting to get money […] they find out they’re pregnant and they go get the abortion, rather than, ‘I didn’t get my abortion yet because I’m saving money or waiting for a paycheck’, or whatever. It’s like, oh, ‘I wanna have my abortion, I’m gonna go have it now, it’s free’.” (TF, abortion provision)
	I know that [clinic name] has felt that they’ve seen earlier patients, because they could have it done earlier. I don’t know if we’ve seen the same thing. (ED, abortion provision)
	I think the other effect that we saw that we were really excited about – again, because it ties back to why we wanted to pass HB 40 – is that we saw a surge in women coming in earlier in the pregnancy, [...]. And I think that’s just proof well, how financial hardships were preventing women from seeking care early on. (DK, abortion provision)
*Patient-driven care (choice of facility)*	"Well, I hope that patients have more access to abortion care in their home institutions and will not have to rely on any place like our county hospital which provided a noble service but it was kind of an assembly line and not very patient-centered." (CL, abortion provision)
	“Now patients with Medicaid are going to have the choice of going to a really private clinic that they can spend the whole hour with just the doctor, or they can have the choice to go to one of the other clinics. But the point being that they have the choice to go and have the service be covered under their own insurance.” (BK, abortion provision) “Now patients with Medicaid are going to have the choice of going to a really private clinic that they can spend the whole hour with just the doctor, or they can have the choice to go to one of the other clinics. But the point being that they have the choice to go and have the service be covered under their own insurance.” (BK, abortion provision) “Now patients with Medicaid are going to have the choice of going to a really private clinic that they can spend the whole hour with just the doctor, or they can have the choice to go to one of the other clinics. But the point being that they have the choice to go and have the service be covered under their own insurance.” (BK, abortion provision)
	“HB 40 has allowed us to provide elective abortion services for people within our system that we hadn’t been able to serve in the past. We have, for example, our resident clinic that sees predominantly Medicaid patients, and a lot of those patients in the past wanted elective terminations and we couldn’t do them, and now all those patients we’re getting in and we take care of them ourselves.” (SP, abortion provision)
	Before House Bill 4, for patients that were self-pay they didn’t have the option of going to the operating room [and getting sedated] for their procedure because the out-of-pocket cost was exponentially higher. But for patients who had Medicaid coverage after HB 40, those patients did have the option of having their procedure in the operating room. (AD, abortion provision)
*Patient-focused care*	Yeah. So that’s actually – I would say in providing – other than the satisfaction which is the top thing – the next best thing – and it’s a good thing – is that I’m more likely now to refer someone for hospital-based care […]: So either in an operating room, or at [names of facilities], where they have a clinic that’s based in the hospital. So if someone coded, or someone couldn’t breathe, you could make a call, and within three minutes have a team of anesthesiologists there, as opposed to at [facility not based at hospital] where we’d have to call 911 (ED, abortion provision)
	“It is completely changing the way that we provide abortions. It’s treating it as just a medical service. It’s not [a] herd of patients showing up at one time [for counseling] [...] So I think in a lot of ways, it’s a very patient-focused style of medicine, and most patients on Medicaid otherwise would not be able to have that service. (BK, abortion provision)
	I mean, the biggest part – the most exciting thing about HB40 was that we could finally see our own patients. We didn’t have to send them off for abortion like it was some dirty thing that we don’t do here at [facility] because we do do it. And this clinic right where we are, where I’m sitting, is the resident clinic, so it’s majority Medicaid patients [...] and we don’t have to turn them away and they get the continuity of care and everything. (GM, abortion provision)
	I would hope that we were doing everything that we could to engage them in ongoing care to get them connected with family planning methods that they would be interested in to prevent subsequent unplanned pregnancies. Or just get them in a primary care. (FK, abortion provision)
	"Now that we take all these Medicaids, we're able to fill those vacancies quicker. So, like say if three people cancel, we're able to find three people who are in need, and we already know that we take their Medicaid. So, I would say because of that and having access to all these Medicaids, our clinic has been able to be a lot more full, a lot more productive, a lot more efficient. So, I would say that's definitely been a plus in that. (BG, abortion provision)
*Removal of discriminatory insurance policy*	“I think that by allowing for Medicaid reimbursement for abortion services, it allows individuals who earn low incomes to have the same access to life-saving services that someone with private insurance would have.” (RA, policy involvement)
	“So, I think it's great that Medicaid is treating abortion like other services that are part of the spectrum of reproductive health care, and not singling it out […] I see this […] coverage as equalizing the program even more.” (FK, abortion provision)
	I think the idea that Illinois would expand its state Medicaid coverage to include abortion care is really important from an equity lens so, that a wide array of people can have access to abortion care regardless of their income. (JN, resource provision)
*Decreased abortion stigma*	I think that the passage of HB40 definitely- definitely moves the needle on the stigma around abortion care, around the rules of who can access abortion and who can't. I think it, it was a huge win, a huge win. (RA, policy involvement)
	No, I think for patients specifically with Medicaid, it definitely – the stigma has reduced, right? Because just knowing that your procedure is covered by your insurance and it’s recognized as part of normal reproductive healthcare that you can go to your reproductive healthcare provider. (GM, abortion provision)
	"I just think it helps with de-stigmatizing it. It's weird that every other reproductive health service is covered under Medicaid and not abortion, you know? Like that sends a really big message right away as to whether or not somebody is allowed to get one, whether its safe, whether - you know? And so I think there's just a lot there as well. (HW, policy implementation)
	“I do see it as a victory because it’s so rare that there’s good public discussion on how abortion should be part of regular medicine. (VS, abortion provision)
Gaps in patient access to abortion	
*Uninsured populations and populations ineligible for Medicaid*	“I think with HB40 it would be really nice if it wasn’t just limited to people on Medicaid. I think it should be anyone who has any insurance or anyone who doesn’t have any insurance, because we know that there’s so many people that are uninsured so that means they’re not on Medicaid or anything.” (RD, resource provision)
	I think documented immigrants, immigrant folks who have access to Medicaid, for sure, they have benefits from it but folks who are undocumented and then also with the new public charge rules, there are a lot of people who are just afraid of what they're allowed to have and are just not enrolling in any support services. So that's the fear - it's like, even if it was covered, I think people wouldn't be keeping it under the shelf like potential public charge later on. (LV, policy implementation)
	“Even if we have Medicaid-funded abortion services, many in our community don’t qualify for Medicaid because of the five-year bar. [...] So even if we repealed Hyde and Hyde was never a thing anymore or not a thing anymore, there’s a significant number of Asian Americans and other immigrants who still would not have affordable access to abortion care because they do not qualify for Medicaid because of the five-year ban.” (HH, policy involvement)
	I mean, always folks that should be eligible but aren’t for Medicaid, mostly undocumented folks, but I think there’s still people that fall through the cracks. So how do you deal with that? And then also like it’s great and this helps a huge number of people, but you look – somebody that’s just over this income limit still doesn’t have that kind of money to cover procedures like that. So I think looking forward to how you get the rest of the folks that are struggling for access still. So a huge step in the right direction, but I think there are still things within the system to improve and then what about the people that fall through the cracks of that system. (FR, policy involvement)
*Minors*	“If you are a young person who is now pregnant, who does not have Medicaid […] you can’t go and just get it on your own and use that to pay for an abortion. Your family needs to do the application for you and with you. And if you don’t want your parents to know that you’re pregnant, you can’t do that.” (HR, policy implementation)
*Rural Populations*	“And so I know that the experience that women who live in this region have in accessing services is much different than somebody in Central or Southern Illinois, where transportation might be a bigger barrier or just finding a willing provider that is within a reasonable distance of where you can get to […] Having a service covered doesn't mean automatically mean access.” (FK, abortion provision)
	But we know in a kind of more general sense that there’s lack of providers and there’s a lack of providers that go all the way to 23.3 weeks in Illinois, outside of Chicago (RD, resource provision)
	"What does it mean, for people, for example, who are living in rural Illinois in the area that used to be served by the [clinic] that's now closed down? Partially as a result of-of reimbursement rates being very low and not being able to, kind of, stem the tide, you know? So in the short term [...] they've lost access, essentially. Whether or not that actually is gonna be a long-term impact now that rates are better and the reimbursement processes are better, I don't know." (HW, policy involvement) "What does it mean, for people, for example, who are living in rural Illinois in the area that used to be served by the [clinic] that's now closed down? Partially as a result of-of reimbursement rates being very low and not being able to, kind of, stem the tide, you know? So in the short term [...] they've lost access, essentially. Whether or not that actually is gonna be a long-term impact now that rates are better and the reimbursement processes are better, I don't know." (HW, policy involvement)
Factors diminishing the impact of HB-40 on individuals seeking abortion care	
*Lack of public knowledge about the law*	"Also think probably that’s partially related to the information not being totally disseminated in the whole system, so – in the whole county really, and everyone knowing that for a long time it was 75 bucks at [facility name] and we haven’t gone – we don’t do any publicity or anything. So no one knows, unless you call and ask specifically that it’s changed, most people come probably expecting that it’s $75, and then are sort of pleasantly surprised that it’s not." (AM, abortion provision)
	“I feel like now it's just like the fact that so many people still don't know […] even if people like are told that their Medicaid was covered [...] they wouldn't believe that [...] because abortion is still stigmatized and politicized that they would expect for it to not be covered [...] It doesn't seem like there are like a lot of resources or like energy behind uh, like spreading awareness rather. Like it's something that [organization name] is trying to do but um, outside of that, it's not like a priority of like the government, you know.” (CJ, resource provision)
	" I think there could be many, many, many more people who could benefit from it if there was more education around it (LV, policy implementation)
	"So again – and then it passed, and then even around implementation and like community education – there’s been no community education done about it. People don’t know about it at all, and all of this talk about implementation has been very insider, like policymakers and insurance experts and Medicaid experts sitting around talking rather than like actually involving community – again, like actually involving people that are impacted by it." (HH, policy implementation)
	[…] people would call the help line who have Medicaid and they're like asking funding and then we tell them if they can just- they go to a different clinic, they can use their Medicaid. But like clinics weren't giving that information to people because they wanted that business essentially. And that was frustrating because, I understand the business, but like our priority is people. Like care and it seemed dishonest and like frankly messed up to like not tell somebody that they could get coverage if they went somewhere else. So-- and that was difficult for us to navigate as funders as well cause we want to maintain relationships with the clinics. (CJ, resource provision)
	“Are there ways to reach out to other providers, like primary care, other OB-GYNs to let them know? Because they don’t even know, in a lot of cases. So it may be that that’s what comes next.” (FR, policy involvement)
*Insurance-related logistical hurdles*	“So there are pregnant women who come in for prenatal care and they’re not enrolled in Medicaid, there’s a presumptive eligibility clause that says okay we’re going to presume you’re going to be paid for this service to provide you early prenatal care, because it might take you a couple of months to get on Medicaid. So they know they will retroactively be paid, so they’re more likely to provide the service [...] if you come in [seeking abortion] and you’re in an early pregnancy stage and you don’t have the money to pay for [the abortion] we say “Well, you have to enroll in Medicaid. Come back in three months after you’re enrolled.” Yeah. I mean so that is ridiculous, right? So it reduces–it increases the risk for the mom. […] It’s just – it’s not okay.” [WW, policy implementation)
	After HB 40, there was some patients we kind of had to sort out insurance information with. So for instance – there were patients that came that thought that they had Medicaid and when we looked them up in the system it was no longer active. There were patients who didn’t think they had Medicaid and it turns out that they did. So there was that component of trying to figure out what their status was – became incorporated into the clinic flow. (AD, abortion provision)
	“The issue of what is often called, in the public benefits field, ‘churning’ is a whole separate issue that affects a lot of low-income people. Which is this concept that we make applying for and remaining on public benefits programs way too complicated, so people are constantly churning on and off these. They’re eligible, then they’re not eligible, then they have to reapply. And so, people are constantly losing eligibility. And that applies in TANF, SNAP, Medicaid, all sorts of public benefit programs.” (HR, policy implementation)
	"But the bottom line of HB-40-it was a major legislative victory but behind the scenes has really not provided the reimbursement necessary for clinics to actually stay open if they start immediately taking Medicaid." (YV, abortion provision)

### Perceived mechanisms connecting increased affordability to abortion utilization

Many participants reported that HB-40 removed a significant financial barrier for Medicaid recipients in need of abortion care, which in turn made abortion more affordable and accessible, expanded patient choice of abortion facilities and methods, and improved the quality of healthcare services by making them more patient-focused.

#### Reduced financial stress

Many thought HB-40 made abortion more affordable for Medicaid recipients, which positively affected the physical and emotional health of patients and their families and may have indirectly made abortion more affordable for patients without Medicaid.

Acknowledging the financial implications of the law for patients, one participant explained, “the vast majority of people are just incredibly grateful that they […] are able to access [abortion] services without a significant amount of difficulty in terms of the cost.” (YV, abortion provision).

Another participant described how insurance coverage of the procedure could reduce the emotional stress some abortion seekers face, by allowing Medicaid patients to use their limited resources on secondary costs needed to access abortion, such as transportation and child-care.

The removal of out-of-pocket costs was thought to benefit not only the health and emotional wellbeing of patients themselves, but also of their families. As described by one participant:

“In the beginning for the first three or four months women would show up because they’d already been scheduled before HB-40 and so they would have their money ready to pay. And we got to tell them that we didn’t need that money. That it was now gonna be covered by their insurance. And so we definitely had people crying and saying how happy they were. And how now they could pay their rent or buy food for their kids.” (OZ, abortion provision)

In addition to the law’s direct impact on Medicaid recipients, a few participants noted the law may indirectly make abortion more affordable for individuals with limited incomes that do not qualify for Medicaid. Participants explained that since individuals with Medicaid now have abortion coverage, there are more financial support resources available for Illinois residents ineligible for Medicaid, as well as for abortion seekers that come to Illinois from states with more restrictive abortion laws. According to one participant,

“It's more like an indirect benefit because […] we'll be able to give a fund to somebody who's undocumented if they didn't have to also give that fund to somebody who could use Medicaid […] so I think there's […] now more resources for people who have less access.” (LV, policy involvement)

Another participant described that after HB-40 their organization “started definitely funding a lot more out-of-state folks […] because [out-of-state patients] couldn’t use [their] form of insurance, [their] Medicaid insurance in Illinois, so they weren’t coming because of HB-40, it just changed the dynamics in how [the organization] funded” (NH, resource provision).

#### Increased patient-driven and patient-focused care

Some participants thought insurance coverage of abortion would provide patients the opportunity to have an earlier medication abortion and allow them to have more choice about where they would like to receive care. In addition to patients having more choice, some also thought the law would lead clinicians to provide patients with higher quality care.

One participant working at an organization involved in reproductive health care policy speculated that patients would be able to obtain abortion care at earlier gestations because they no longer had to spend time gathering money for the procedure. This theory was substantiated by another participant who described her abortion facility as experiencing “a huge increase in medical abortions”, which is offered up to 10 weeks gestation. This participant further explained,

“I think it’s because of Medicaid. They’re not waiting to get money […] they find out they’re pregnant and they go get the abortion, rather than, ‘I didn’t get my abortion yet because I’m saving money or waiting for a paycheck’, or whatever. It’s like, oh, ‘I wanna have my abortion, I’m gonna go have it now, it’s free’.” (TF, abortion provision)

Another participant stated,

“We saw a surge in women coming in earlier in the pregnancy, […]. And I think that’s just proof well, how financial hardships were preventing women from seeking care early on.” (DK, abortion provision)

Some participants also thought HB-40 could result in patients having a wider range of abortion facilities from which to choose. According to one participant,

“Now patients with Medicaid are going to have the choice of going to a really private clinic that they can spend the whole hour with just the doctor, or they can have the choice to go to one of the other clinics. But the point being that they have the choice to go and have the service be covered under their own insurance.” (BK, abortion provision)

However, a few participants highlighted that, in some cases, patients may not be able to have their abortion at their preferred facility if that facility does not accept a patient’s Medicaid plan. One provider at a clinic that accepted a limited number of Medicaid plans that covered abortion under certain circumstances explained:

“If you have those three plans that we currently accepted just for the specific indication, we now can accept it and they will pay for your abortion. But any other Medicaid plans we have to turn away […].And so when [patients] call and schedule […] I think that’s a surprise to them. And sometimes it’s a happy surprise whereas they don’t have to pay the money and they can go somewhere else. And sometimes they’re upset that they can’t have the procedure done where they would prefer to have the procedure.” (OZ, abortion provision)

A few participants explained how HB-40 improved the quality of care for Medicaid recipients because it resulted in more patient-focused care. One participant working at a facility that kept costs low for patients by maintaining a fast pace for clinic flow, described the change in quality of care after the passage of HB-40:

“It is completely changing the way that we provide abortions. It’s treating it as just a medical service. […]So I think in a lot of ways, it’s a very patient-focused style of medicine, and most patients on Medicaid otherwise would not be able to have that service.” (BK, abortion provision)

Some participants also discussed how HB-40 led to increased opportunities for ensuring continuity of care. According to one participant who worked at a facility that previously could only provide abortion care to Medicaid patients eligible via the circumstances outlined by the Hyde Amendment:

“I mean, the biggest part – the most exciting thing about HB-40 was that we could finally see our own patients. We didn’t have to send them off for abortion like it was some dirty thing that we don’t do here at [facility] because we do do it. And this clinic right where we are, where I’m sitting […] it’s majority Medicaid patients [...] and we don’t have to turn them away and they get the continuity of care and everything. (GM, abortion provision)

Another participant emphasized the value of connecting abortion care to additional engagement in contraceptive counseling and primary care:

“I would hope that we were doing everything that we could to engage [patients] in ongoing care to, get them connected with family planning methods that they would be interested in to prevent subsequent unplanned pregnancies. Or just get them in a primary care.” (FK, abortion provision)

#### Removal of discriminatory insurance policy and decreased abortion stigma

A few participants discussed how HB-40 removed a discriminatory insurance coverage policy, and some thought equitable insurance coverage of abortion might contribute to a reduction in abortion stigma. As one participant shared,

“I think the idea that Illinois would expand its state Medicaid coverage to include abortion care is really important from an equity lens so that a wide array of people can have access to abortion care regardless of their income.” (JN, resource provision)

Another participant described how HB-40 made access more equitable for individuals earning low-incomes:

“I think that by allowing for Medicaid reimbursement for abortion services, it allows individuals who earn low incomes to have the same access to life-saving services that someone with private insurance would have.” (RA, policy involvement)

Although not all privately insured people experience coverage for abortion care, this participant’s comment illustrates the general perception among interviewees that HB-40 improved equity in insurance coverage for abortion care, especially since in 2019, Illinois began requiring state-governed insurance plans to provide abortion coverage if they offered maternal health coverage.

Some participants thought making abortion “a part of regular medicine” by including it as a covered service would contribute to a decrease in abortion stigma. According to one participant:

"I just think it helps with de-stigmatizing it. It's weird that every other reproductive health service is covered under Medicaid and not abortion, you know? Like that sends a really big message right away as to whether or not somebody is allowed to get one, whether it’s safe, […] ” (HW, policy involvement)

## Perceptions of persistent barriers to utilization

Although participants viewed HB-40 as having an overwhelmingly positive impact on patients, many highlighted specific groups of patients that continue to experience difficulties accessing abortion care. Several participants pointed out that recent immigrants and undocumented individuals are not eligible for Medicaid, so would not directly benefit from HB-40. Comments from participants highlight the desire for Medicaid to cover a wider swath of individuals, as well as the need to address all policies that affect access to Medicaid, such as the policy preventing recent immigrants from enrolling in Medicaid for five years. According to one participant:

“I think with HB-40 it would be really nice if it wasn’t just limited to people on Medicaid. I think it should be anyone who has any insurance or anyone who doesn’t have any insurance, because we know that there’s so many people that are uninsured […]g.” (RD, resource provision)

Another participant shared a perspective on patients their organization served, including immigrants who could not immediately access Medicaid:

“Even if we have Medicaid-funded abortion services, many in our community don’t qualify for Medicaid because of the five-year bar. […]So even if we repealed Hyde and Hyde was never a thing anymore or not a thing anymore, there’s a significant number of Asian Americans and other immigrants who still would not have affordable access to abortion care because they do not qualify for Medicaid because of the five-year ban.” (HH, policy involvement)

Another participant thought even immigrants who do qualify for Medicaid may be afraid to enroll because they believe it could lead them to be considered a “public charge”, which could threaten their ability to obtain permanent residency in the future.

Other groups that participants thought might not benefit from HB-40 included young people and people living in rural parts of the state. A few participants discussed how young people under the age of 18 may still find accessing abortion difficult. As one participant stated:

“If you are a young person who is now pregnant, who does not have Medicaid […] you can’t go and just get it on your own and use that to pay for an abortion. Your family needs to do the application for you and with you. And if you don’t want your parents to know that you’re pregnant, you can’t do that.” (HR, policy involvement)

Some participants talked about how for people living in rural parts of the state, it may be difficult getting to an abortion provider. As one participant shared:

“And so I know that the experience that women who live in this region have in accessing services is much different than somebody in Central or Southern Illinois, where transportation might be a bigger barrier or just finding a willing provider that is within a reasonable distance of where you can get to […] Having a service covered doesn't automatically mean access.” (FK, abortion provision)

Finally, one participant at an organization involved in the passage of HB-40 thought people earning just above the income threshold to qualify for Medicaid and people with private insurance who have high deductibles or co-pays may also continue to encounter financial barriers to accessing abortion.

## Factors that could diminish the impact of HB-40 on abortion care affordability

Participants described two factors related to policy implementation that could dull the impact of HB-40 on patients seeking abortion care: a lack of public knowledge about the law and insurance-related logistical barriers.

### Lack of public knowledge about the law

Many participants involved in policy efforts and resource provision reported that a lack of public knowledge about Medicaid coverage of abortion was a barrier to access. One participant thought “there could be many, many, many more people who could benefit from it if there was more education around it” (LV, policy involvement). Another participant did not think abortion seekers benefited at all from HB-40 at the time of the interview “mostly because people don’t know about it.” This participant further explained:

“There’s been no community education done about it. People don’t know about it at all, and all of this talk about implementation has been very insider, like policymakers and insurance experts and Medicaid experts sitting around talking rather than like actually involving community – again, like actually involving people that are impacted by it.” (HH, policy involvement)

The importance of public education campaigns was highlighted by another participant who thought “even if people are told that their [abortion] was covered […] they wouldn’t believe that […] because abortion is still stigmatized and politicized that they would expect for it to not be covered.” (CJ, resource provision). In particular, one participant thought efforts should be made to educate a wider array of providers about the new coverage available after the passage of HB-40:

“Are there ways to reach out to other providers, like primary care, other OB-GYNs to let them know? Because they don’t even know, in a lot of cases. So it may be that that’s what comes next.” (FR, policy involvement)

Although many participants working at abortion facilities reported discussing Medicaid coverage with individual patients who called seeking care, they did not necessarily employ broader outreach efforts. As one provider explained:

“At least once a day someone will ask, ‘Well, so do I pay now or when do I pay for this or – so we’re not supposed to pay for this anymore?’ So there is some confusion still about that […] I also think probably that’s partially related to the information not being totally disseminated in the whole system, so – in the whole county really […]. So no one knows, unless you call and ask specifically that it’s changed, most people come probably expecting that it’s [amount], and then are sort of pleasantly surprised that it’s not.” (AM, abortion provision)

At the same time, a few participants from community-serving organizations voiced concern over whether abortion facilities were providing full information on Medicaid coverage. One participant described how some clinics that did not accept Medicaid “weren’t giving that information [about Medicaid coverage of abortion] to people because they wanted that business”, which this participant thought was “dishonest and like frankly messed up to not tell somebody that they could get coverage if they went somewhere else” (CJ, resource provision).

### Insurance-related logistical hurdles

Participants discussed a need to improve insurance-related processes that made it difficult for patients to enroll in Medicaid and retain coverage and made it hard for facilities to provide care to Medicaid recipients. Two examples of these hindrances were the inability of abortion clinics to offer presumptive eligibility to patients and the low Medicaid reimbursement rates for abortion care.

A few participants described the impacts of Medicaid’s complicated enrollment system and eligibility requirements on patients. As one participant explained:

“The issue of what is often called, in the public benefits field, ‘churning’ is a whole separate issue that affects a lot of low-income people. Which is this concept that we make applying for and remaining on public benefits programs way too complicated, so people are constantly churning on and off these. They’re eligible, then they’re not eligible, then they have to reapply. And so, people are constantly losing eligibility. And that applies in TANF, SNAP, Medicaid, all sorts of public benefit programs.” (HR, policy involvement)

Some also discussed the need to adjust state requirements to allow abortion clinics to qualify to bill for care for Medicaid-eligible participants before they are enrolled in Medicaid [8]. This process, known as Medicaid presumptive eligibility, is critical for ensuring that patients who qualify for Medicaid receive timely care “because it might take [patients] a couple months to get on Medicaid.” The consequence of which, is the abortion patient not receiving care when they want it or in some cases not receiving the preferred abortion care.

In the immediate wake of HB-40’s implementation, low Medicaid reimbursement rates and complicated reimbursement processes were highlighted as a major concern for the sustainability of abortion facilities in the state. Many participants worried that low Medicaid reimbursement rates would force some abortion clinics to close, which would ultimately decrease abortion access for Medicaid patients. According to one participant, HB-40 “has not provided the reimbursement necessary for clinics to actually stay open if they start immediately taking Medicaid” (YV, abortion provision). In addition to clinic closures, one participant described that low reimbursement rates made their facility reluctant to accept more Medicaid plans since it was easier and more sustainable to “just keep seeing the [patients with] private insurances that we take, patients who have no insurance that pay in cash and [patients with] Medicaid plans that we know that we’ve already had contracted with […] it’s not a new big revenue stream to see these patients who have Medicaid that we don’t take.” (OZ, abortion provision). Although it is unclear how many facilities accepted all Medicaid plans after HB-40, the inability of clinics to do so may have prevented some patients from fully benefiting from HB-40. Following an increase in Medicaid reimbursement rates in December 2019, participant narratives about reimbursement shifted from discussions about sustainability of abortion services to the need for clarity or training on the steps abortion facilities need to take to receive reimbursements. Reimbursement challenges and processes are described in greater detail in another paper [10].

## Discussion

This study’s findings on the perceived benefits of Medicaid coverage for some patients seeking abortion complements previous studies documenting how out-of-pocket costs or lack of insurance coverage delay or prevent patients from receiving abortion care [11, 15–18]. Research has shown how difficulties paying for an abortion lead individuals to delay or forego payments for rent, utilities, and food [3, 12]. Some individuals have no choice but to carry an unwanted pregnancy to term because of the inability to pay for an abortion, which has implications for long-term economic outcomes [19]. Participants in our study perceived that removing a significant financial barrier, made abortion care more affordable for individuals with limited resources, made it possible for patients to allocate their limited income to cover secondary costs to obtaining an abortion or to other essential household needs, and could result in patients seeking abortion care earlier in their pregnancy. These perceptions support results from a recent study in Oregon, which found that expansion of the state’s Medicaid program (which covers abortion care) was associated with an increase in Medicaid-financed abortions as well as an increase in medication abortions among patients using Medicaid for their abortions [20]. Our results also build upon previous studies about the impact of Medicaid coverage of abortion in two ways. First, our results suggest that expanded Medicaid coverage of abortion may indirectly benefit patients without insurance coverage because it frees up resources that would have been allocated to patients eligible for Medicaid. Second, our findings reveal how Medicaid coverage alone may not address all gaps in abortion access and highlights the need to address non-financial barriers created by existing laws, policies, or programs.

Findings from our study highlight certain populations that may not benefit from HB-40 due to their citizenship status, age, or location of residence. Following implementation, participants were concerned that recent and undocumented immigrants in particular would face unique challenges to obtaining abortion care. In Illinois, people considered “qualified non-citizens”, such as lawful permanent residents, must wait 5 years before they can enroll in Medicaid [21]; however pregnant patients may be enrolled in Medicaid regardless of immigration status for the duration of their pregnancy. Post data collection, Illinois expanded the category of providers who could offer presumptive eligibility enrollment for pregnant patients, allowing qualified non-citizens to obtain coverage for abortion care in addition to prenatal care, labor, and delivery.

The federal government’s public charge rule – which defined a non-citizen as a “public charge” if they are currently or expected to become dependent on certain publicly funded programs – was expanded in 2020 to include the use of more programs, including federally-funded Medicaid. If deemed a “public charge”, a person is ineligible to attain lawful permanent residence if applying for a green card. Although the revised public charge rule did not include Medicaid used by pregnant people and does not apply to state-Medicaid funds, the law’s intent to bar immigrants from receiving public assistance could incite fear and confusion about which public programs they can utilize without jeopardizing their chance to remain in the US or obtain permanent residence [22]. Leading medical organizations issued a statement opposing the changes to the public charge policy, stating it threatens patient health and leads to more complex public health challenges [23]. Although the Department of Homeland Security announced the revised public charge rule is no longer in effect [24], public education campaigns informing immigrant communities about the current public charge rule could help reduce confusion and ensure that all abortion seekers with Medicaid can use their insurance for their abortion without fear.

Abortion seekers residing in rural areas of Illinois may also continue to encounter challenges that delay or prevent them from receiving abortion care. Previous research has shown an association between increased distance to a provider and a decrease in abortion rates [25, 26]. Expenses related to travel - such as gas, hotel stays, and childcare costs - combined with lost wages from taking time off of work, could delay or prevent many abortion seekers from getting to a clinic in the first place [3]. Although Medicaid may cover transportation costs to abortion appointments in Illinois, coverage is determined on a case-by-case basis [27]. Providing clear information about Medicaid coverage of transportation costs, as well as information about abortion funds or other resources that may be able to provide financial assistance for travel-related expenses for patients ineligible for Medicaid, will benefit abortion seekers in the state.

Our results also touched upon the relationship between legality and stigma, with some participants perceiving that legally requiring insurance coverage of abortion affirms that abortion is an essential part of reproductive health care, which could lead to a reduction in abortion stigma. Although additional research is needed to assess the impact of abortion laws like HB-40 on views held by abortion seekers and the general public, previous studies have documented the negative financial and health impacts of stigma on abortion patients. One study found that perceived stigma contributed to patients’ decision to not use their insurance to cover their abortion [12], and two studies reported a strong association between perceived abortion stigma and poor psychological wellbeing before [28] and after having an abortion [29]. These studies suggest that reducing stigma could result in better mental and financial health outcomes for abortion patients. However, opinions that HB-40 helps reduce stigma rest on the assumption that patients are aware of the law, and many participants thought a lack of public education around HB-40 prevented the law from increasing abortion access to the extent it could have. Further research is needed on the relationship between abortion stigma, abortion-related laws and policies, and the experiences of individuals who want or have had an abortion.

## Limitations

Although this study interviewed a diverse set of participants well positioned to describe general trends and changes in abortion provision and receipt, we did not speak directly with abortion patients about Medicaid coverage of abortion and its impact on their abortion-seeking experiences. A deeper understanding of abortion seekers’ knowledge of and thoughts about the law could highlight the extent to which the law affected their decision-making process, as well as their experiences seeking or obtaining abortion care. As a next step, members of our research team have a separate study underway to explore patient perspectives on insurance coverage for abortion in Illinois and its effect on their abortion experience.

A second limitation was the 12-month time span in which team members conducted interviews, as participants’ views about the effects of and experiences with HB-40 may have shifted over time. For example, participants in earlier interviews often cited concerns that patient access would be limited in the long-term if clinics are forced to close due to low Medicaid reimbursement rates, but this concern was not raised in later interviews, following increases in the Medicaid reimbursement rates in December 2019.

A third limitation was that the interview guide for providers and administrators working at abortion facilities was different from the guide used when interviewing staff from other organizations, which may have impacted when and how topics were raised during interviews. For example, lack of knowledge about HB-40 was described as a barrier for patients by non-providers, whereas this same topic was brought up by clinic staff when describing how surprised patients were when finding out that Medicaid would cover their abortion. This difference may have stemmed from the fact that participants not working at facilities providing abortion care were asked about the impact of the law on people seeking abortion in Illinois, whereas providers were asked about the law’s impact on their patients and facility in particular.

In addition, all except the final 5 interviews were conducted before the COVID-19 pandemic was declared a national emergency on March 13, 2020, and the pandemic’s wide-ranging impacts on healthcare access was a topic we did not discuss in interviews. Future research could address this by exploring how cost and insurance barriers intersect with other factors affecting physical and virtual access to abortion care going forward.

## Conclusion

In the first year of implementation of the policy, HB-40, abortion providers and others who provide support for abortion services and/or were involved in the passage of HB40 reflect that Medicaid coverage of abortion seemed to reduce financial stress and improve the experience of seeking abortion care for patients in Illinois. Further, expanding Medicaid coverage to abortion care was perceived to normalize abortion care as a basic reproductive health care service and to have a positive impact on resource shifting—patients with Medicaid coverage can allocate finances to other needs and more financial support can be directed towards patients without insurance coverage. However, significant obstacles remain for certain groups, including minors, undocumented immigrants, and people residing in rural areas. Further evaluation of the legal barriers for immigrants and the challenges associated with Medicaid enrollment and awareness of insurance coverage of abortion are needed to fully understand the impacts of these policies on abortion access.

## Supplementary Information

**Additional file 1.** Interview Guides used during Provider and Non-Provider Interviews.

## Data Availability

The data analysed during the current study are not publicly available because it would infringe on the confidentiality agreement we have made with study participants. Research instruments can be shared upon request to the corresponding author.

## Abbreviations

CHIP: Children’s Health Insurance Program
HB-40: House Bill 40
OB-GYN: obstetrician-gynecologist
SNAP: Supplemental Nutrition Assistance Program
TANF: Temporary Assistance for Needy Families
