# Supplementary material for: Provider and community stakeholder perspectives of expanding Medicaid coverage of abortion in Illinois
Source: BMC Health Serv Res. 2022 Mar 28;22:413. doi: 10.1186/s12913-022-07761-5 (PMC8960679; doi:10.1186/s12913-022-07761-5)
Supplement: Supplementary file 1 — Additional file 1. Interview Guides used during Provider and Non-Provider Interviews. [file 12913_2022_7761_MOESM1_ESM.pdf]

Understanding Abortion Care Provision before and after Illinois Policy Change
In-Depth Interview of Providers

Study ID:	
Interviewer:	
Date:	
	Turn on voice recorder and check that it is working.
Has this participant given verbal consent?	Yes No (check consent form, circle one)
	Inform participant that the call is now being recorded.
	Say the participant's study ID number so it's on the audio record.
Time recorder turned on:	___________ am/pm

Thank you for taking the time to speak with me today. We are trying to understand your perspective and experience before and immediately after HB40 was passed in Illinois. Participation is voluntary and there is no penalty for refusing to participate. Additionally, the survey will not include any information that could be used to identify you. As a token of our appreciation, we will offer you a $60 gift card at the end of the interview. We may also ask if you have a colleague you think would be particularly appropriate for us to speak with, given the aim of the study.

Interviews will take 60-90 minutes to complete. Interviews will be audio-taped and securely stored. Your identity will not be shared with anyone outside our research group, which includes Aalap Bommaraju, and researchers at Ibis Reproductive Health and the University of Chicago.

Are you interested in participating?

Do you have any questions about I have just explained before we begin?

[If yes] *Great, I will turn on my recorder.*

[If no] *No problem, have a great day.*

1 Participant Information

First, I'd like to ask some general background questions about you.

1.1 How many years have you been involved in the provision of abortion services?

1.2 How many years have you been involved in abortion provision <u>at this practice or organization</u>?

1.3 What is your role in the provision of abortion services at this clinic or organization?

2 Insurance Coverage

Now we're going to talk about the effect of HB 40 on the services you provide at your clinic. I'll ask you a question and I'd like you to tell me about how things worked before HB 40 and how they work now (after HB 40).

2.1 Thinking about all kinds of insurance, including Medicaid and private insurance, did you accept any insurance for abortion care before HB 40?
Probe: What kinds?

And after HB 40?

If participant indicates they do not currently accept insurance coverage, skip to question 2.8

2.2 Before HB 40, what was the average reimbursement your clinic received for abortions? For Medicaid? For private insurance?

After HB 40, what is the average reimbursement your clinic receives for abortion? For Medicaid? For private insurance?

Probe:
Can you describe the billing codes you use for different kinds of abortion services?

2.3 Before HB 40, were there any Medicaid plans that you tried to establish a relationship with but were unable to?

If YES, probe:
What challenges did you encounter in trying to establish a relationship with them?

After HB 40, has the situation changed or stayed the same?

If changed: How have things have changed?

2.4 Before HB 40, can you describe your experience receiving reimbursement for abortion services from Medicaid and Private insurance companies that you worked with?

Probe:
What things have made it easy or difficult to receive reimbursement services from the insurance company(ies) that you worked with before HB 40?

Again, thinking about the situation before HB 40: How long did it usually take to receive reimbursement for abortion services from Medicaid?

Probe:
Do you recall any cases being approved or paid for quickly? What do you believe were the reasons for this to happen quickly?

Can you describe any instances when approval or reimbursement took longer than usual? What do you believe led to a delay?

Did you notice any patterns? For example, was there a difference in timing by procedure?

If participant accepts multiple types of plans, probe:

Have you noticed any differences in timing by insurance plans, i.e., Medicaid v. private plans?

How does your experience compare with your reimbursement experience for non-abortion services?

After HB 40, what has your reimbursement experience for abortion services been like?

Probe:

Has it become easier or more difficult to obtain Medicaid reimbursement for abortion services, or has there been little change?

How does your experience now compare with your reimbursement experience for non-abortion services?

2.5 Before HB 40, were there any factors, other than reimbursement, that made it easy or difficult to accept insurance to cover abortion services?

Probe:

Are there any specific challenges or facilitators such as claim processing, out-of-network provider status, prior authorization, or determining eligibility that you've encountered?

Have you ever submitted the same claim to Medicaid multiple times?

If YES, What caused you to submit the claim multiple times? What was the result of the resubmission(s)?

Were any cases ever denied outright by Medicaid?

If YES, Before or after prior authorization? Both? Tell me about what happened.

If participant accepts multiple types of plans, probe:

Have you noticed any differences in these challenges/facilitators between different types of plans, i.e., Medicaid v. Private plans?

After HB 40, what has your experience been like?

2.6 Before HB 40, how did your clinic respond in cases of funding delay or denial for abortion services?

Probe:

Did your clinic absorb the cost?

How often did your clinic absorb the cost?

For which patients, or under which circumstances, was your clinic most likely to absorb costs?

After HB 40, has this response changed or stayed the same?

If changed, Tell me how things have changed?

2.7 After HB40, have you noticed any change in the number of patients, or type of patient questions/experiences seeking insurance coverage for abortion?

If YES: Please describe any changes you've observed

Probe:
Have minors been impacted by the passage of HB 40?

2.8 *[If participant indicates they do not accept any insurance]* What made your clinic decide not to accept insurance coverage for abortions? How long has this policy been in place?

2.9 [*If participant indicated that the clinic accepts Medicaid]* How and when did the clinic first acquire Medicaid certification?
Probe:
How long has your clinic had this certification?

What is your understanding of the requirements to remain as a Medicaid provider in Illinois?

3 Public Funding for Abortion

Now I'm going to ask you some specific questions about your interactions with Medicaid Managed Care Plans and Illinois Department of Healthcare and Family Services.

3.1 Are patients able to sign up for Medicaid at your clinic?
If YES, probe:
Can you describe the sign-up process at your clinic?

Do you offer presumptive eligibility?

If NO, probe: Are you able to provide referrals for patients to apply for Medicaid? Please share your experience in providing referrals.

3.2 When patients call to make an appointment, are they asked if they have Medicaid?
If YES probe: How are patients asked about their Medicaid status? Is this documented anywhere in clinic records?

If NO, probe: Are there other ways that you find out if a patient has Medicaid or not?

3.3 What kinds of questions do patients ask about Medicaid coverage for abortions?
Probe:
What is their response to the information you give them about Medicaid coverage?

3.4 What forms are necessary for approval of Medicaid funding for abortions?
Probe:
Is a signature required on these forms?

Who has to sign the form?

How does the person signing feel about signing their name to the form?

3.5 Can you describe your clinic's current relationship with the Illinois Department of Family Services—the state institution that authorizes Medicaid payment for patients?
Probe:
Have you ever encountered any statements for or against abortion from anyone from this agency? *Probe for narrative*.

3.6 Do you need pre-authorization to see a patient paying with Medicaid?

If YES, probe: How long does it usually take to get authorization for these cases?

After HB 40, have things changed or stayed the same?

4 Making Sense of HB 40's Passage

4.1 What steps has your clinic taken to incorporate the changes needed to comply with HB40?

Probe:

What changes, if any, still need to be made?

Who is responsible for making sure those things happen?

4.2 What factors, if any, have facilitated the translation of HB40 into practice at your clinic?

Probe:

Have you received grants to support the costs of providing services to low-income patients?

What are the conditions you have to meet for these grants?

Have you received technical assistance?

Can you describe what that technical assistance has entailed?

4.3 What factors, if any, impeded or currently impedes the translation of HB40 into practice at your clinic?

4.4 Were you involved with the effort to get HB 40 passed in 2017?

If YES, probe: Tell me about what you did.

4.5 When did you find out about HB 40? How did you learn about it?

4.6 When did you find out that HB 40 was going to be signed into law by the governor? How did you feel about it then?

4.7 How do you feel now about the passage of HB40?

Probe:

Who do you think stands to benefit from HB40?

What do you think they stand to gain from it?

Do you think your patients will benefit from the passage of HB40? Why or why not? Can you give an example of a patient's experience?

Do you think your organization will benefit from the passage of HB40? Why or why not?

Thinking about the changes you have made/are making due to HB40, are there any gaps or issues you wish HB40 addressed that would help your clinic increase access to abortion?

4.8 What do you think the consequences of HB40 will be?

Probe:

For patients?

For providers?

For clinics?

For politics?

For future policy changes that affect abortion access?

5 Conclusion

5.1 Which organizations or persons come to mind when you think about who influences reproductive health policy in Illinois? What about in the Midwest? What about nationally?

5.2 Have you heard about the current effort to change abortion policy in Illinois?

Probe:

What have you heard about this effort?

Do you think that it will impact your organization's ability to provide abortion services in Illinois? How so?

The Reproductive Health Act (HB 2495) establishes the fundamental right to reproductive health which includes right to use/refuse contraception and the right to continue a pregnancy and give birth or have an abortion.

The Reproductive Health Act repeals the Illinois Abortion Law of 1975, which imposes criminal penalties targeting health care providers offering abortion care, and enacts legislation that regulates abortion like any other form of health care. It also requires private insurance coverage for abortion care.

Senate Bill 1594 would repeal the law requiring providers to notify an adult family member before a minor seeks abortion.

5.3 Can you provide us with some demographic information about yourself?

Age	
Sex/gender	
Race/ethnicity	
Sexual orientation	
City, town, village of residence	

We can't interview every abortion provider, including hospitals, in your state, but would like to find those who would be able to share their experience providing care before and after HB 40. Can you recommend other people to interview?

Name	Organization	Contact Info

Thank you so much for your time and willingness to participate.

	Turn off recorder.
Time recorder turned on:	___________ am/pm
Note best email address to send payment to recipient (but do not record audio)	

Understanding Abortion Care Provision before and after Illinois Policy Change
In-Depth Interview of Non-Providers

Study ID:	
Interviewer:	
Date:	
	Turn on voice recorder and check that it is working.
Has this participant given verbal consent?	Yes No (check consent form, circle one)
	Inform participant that the call is now being recorded.
	Say the participant's study ID number so it's on the audio record.
Time recorder turned on:	___________ am/pm

Thank you for taking the time to speak with me today. We are trying to understand your perspective and experience with HB40, passed in 2017 in Illinois. Participation is voluntary and there is no penalty for refusing to participate. Additionally, the study will not include any information that could be used to identify you. As a token of our appreciation, we will offer you a $60 gift card at the end of the interview. We may also ask if you have a colleague you think would be particularly appropriate for us to speak with, given the aim of the study.

Interviews will take 60-90 minutes to complete. Interviews will be audio-taped and securely stored. Your identity will not be shared with anyone outside our research group, which includes Aalap Bommaraju, and researchers at Ibis Reproductive Health and the University of Chicago.

6 Participant Information

First, I'd like to ask some general background questions about you and your role.

6.1 Could you tell me a little bit about what led you to work at [NAME OF ORGANIZATION] and how you got your position here?
6.2 How many years have you been working with your organization?
6.3 How would you describe your role in your organization?
6.4 Can you provide a general description of the work that your organization does to support abortion access in Illinois?

Probe: Does your organization work directly with people seeking abortion?
If yes, Tell me about you/your organization's work with people seeking abortion care
Tell me about your role within that work?

1.5 What is [NAME OF ORGANIZATION]'s role in shaping Illinois' reproductive health policy?

Between 2017 and now, Illinois has passed two major pieces of legislation that have addressed abortion. We'd like to ask you about your impressions of HB40, the legislature signed into law by Governor Rauner in 2017 and implemented in 2018. We also want your perspectives on what the law does and how it has affected you and your organization.

7 General Questions about Recent Legislation

7.1 How did your organization learn about HB40?
Probe: When did you find out about HB40?

7.2 When did your organization find out that HB40 was going to be signed into law by the governor?
Probe: How did you feel about it then? How did others in your organization feel? Did your organization issue a formal response? If yes, what was the organizational response?

7.3 Can you describe your organization's involvement with the effort to get HB40 passed in Illinois?
Probe: Were you involved with the effort to get HB40 passed?
Can you describe your personal involvement?

7.4 What is your current impression of what HB40 is intended to do?

8 Current impressions of HB40

8.1 How do you feel about the passage of HB40 now?
Probe:
Who do you think has benefitted from HB40?

What do you think they have gained from it?

Do you think people seeking an abortion in Illinois have benefitted from the passage of HB40? Why or why not?

Has your organization benefitted from the passage of HB40? Why or why not?

8.2 Are there any gaps or issues you wish HB40 addressed that would help clinics increase access to abortion?

Probe:
What are your thoughts about the reimbursement rates?

What are your thoughts about the certification process for abortion providers?

What are your thoughts about the enrollment and retention process or Medicaid recipients?

What are your thoughts about the claims process?

In your opinion, are there some clinics who may not benefit from HB40?

Have you encountered clinics that do not tell Medicaid patients that they qualify for covered services? Why do you think this has happened?

8.3 Are there any gaps or issues you wish HB40 addressed that would make it easier for people seeking abortions to gain access to services?

8.4 What recommendations would you make to improve the implementation of HB40?

8.5 Has there been a shift in your organization's activities (related to abortion care, access, or policy) shifted since the passage of HB40? *If yes*, please describe those shifts
Probe: Are there new roles for you/your organization that have emerged?
How have they impacted your work with people seeking abortion care?
How have they impacted your work with abortion providers?

8.6 In your opinion how well has HB40 been implemented?
Probe:
Can you recap the main challenges for clinics?
Can you recap the main challenges for people seeking abortion care?
Can you recap success stories?

8.7 What do you think the long-term consequences of HB40 might be?
Probe:
For people seeking abortions?
For abortion providers?
For abortion clinics?
For politics?
For future policy changes that affect abortion access?

9 Other changes since HB40 has been in effect: Questions about the Reproductive Health Act

In spring of 2019, the Reproductive Health Act was passed and signed by Governor Pritzker. This law may have effects on abortion care and access in the post-HB40 setting. Among other things, the Reproductive Health Act requires private insurance companies to cover abortion care in the same way they cover all other pregnancy-related care (e.g., same co-pays, etc.).

9.1 How do you feel about the passage of Reproductive Health Act? How does your organization feel about the Reproductive Health Act?

9.2 How do you think the Reproductive Health Act's requirement for private insurance coverage of abortion will impact patients? Clinics?

9.3 What do you think would be helpful to assist with implementation of the Reproductive Health Act's requirement that private insurance companies cover abortion care?

10 Conclusion

10.1 Which organizations or persons come to mind when you think about who influences reproductive health policy in Illinois? What about in the Midwest? What about nationally?

Probe: Is your organization a part of these larger efforts? *If yes,* what role is your organization playing?

10.2 Can you provide us with some demographic information about yourself?

Age	
Sex and/or gender	
Race and/or ethnicity	
Sexual orientation	
City, town, village of residence	
